# Association between aerobic fitness and attentional functions in Egyptian preadolescent children

**DOI:** 10.3389/fpsyg.2023.1172423

**Published:** 2023-07-06

**Authors:** Osama Abdelkarim, Mohamed Aly, Noha ElGyar, Amira M. Shalaby, Keita Kamijo, Alexander Woll, Klaus Bös

**Affiliations:** ^1^Faculty of Physical Education, Assiut University, Assiut, Egypt; ^2^Institute of Sports and Sports Science, Karlsruhe Institute of Technology, Karlsruhe, Germany; ^3^Faculty of Liberal Arts and Sciences, Chukyo University, Nagoya, Japan; ^4^Faculty of Medicine, Department of Pediatri, Assiut University, Assiut, Egypt

**Keywords:** cognitive control, alerting, orienting, childhood, aerobic fitness

## Abstract

Growing evidence indicates that culture and education can influence cognitive constructs. Studies targeting Western and Asian populations have shown a positive relationship between aerobic fitness and cognitive control in children; however, this association has yet to be explored in the Arab world. The current study aimed to investigate the relationship between aerobic fitness and attentional networks in Egyptian preadolescent children. In total, 103 preadolescent children (9.76 ± 0.11) completed an assessment of aerobic fitness using a 6-min running test and a computerized attention network test that allowed for assessing alerting, orienting, and executive networks. The results revealed that higher aerobic fitness was associated with shorter response time and higher response accuracy in a more cognitively demanding task condition (i.e., incongruent trials). Furthermore, higher aerobic fitness was associated with a more efficient executive network. No associations were observed for alerting and orienting networks. These findings corroborate growing evidence indicating the importance of aerobic fitness for cognitive development and extend the literature by suggesting that the positive association between aerobic fitness and cognitive control might be generalized to the Arab population and not significantly change across cultures.

## 1. Introduction

The increasingly sedentary lifestyle of children has become a prominent concern, with more time spent on screens and less on physical activity. This lack of movement has led to a decline in overall fitness levels, resulting in an increased risk of health issues, such as cardiovascular disease and high blood pressure, which were previously considered adult-onset diseases but are now common in childhood (Cioana et al., 2021). The WHOpredicts that this trend will continue, with an estimated 167 million people becoming overweight or obese by 2025 (WHO, 2022). In Egypt, 20 and 10.7% of children are classified as overweight and obese, respectively, revealing the impact of this lifestyle shift (Talat and El Shahat, 2016; Abdelkarim et al., 2020). The reduction in physical activity is a trend also observed in the Middle East and North Africa (Chaabane et al., 2020). The decline in fitness levels has a negative impact not only on physical health but also on cognitive health (Etnier et al., 2006).

Research has focused particularly on the association between aerobic fitness and cognitive performance in children because childhood is a critical period for brain development (Chaddock et al., 2011). For example, studies have shown that higher fitness levels are linked to better academic performance (Muntaner-Mas et al., 2022) and improved ability to perform cognitive tasks requiring variable cognitive demands (Kamijo and Masaki, 2015, 2016; Kao et al., 2017). For that reason, a growing body of literature from Western and Asian cultures has examined the association between aerobic fitness and cognition in children. Specifically, it has been shown that aerobic fitness is associated with better performance on tasks that tap into cognitive control (Donnelly et al., 2016; Van Waelvelde et al., 2020), which refers to the ability to regulate and coordinate cognitive processes to achieve a goal or task (Miyake et al., 2000). Research has demonstrated that children with higher levels of aerobic fitness tend to have better performance on tasks that measure inhibitory control (Hillman et al., 2009; Pontifex et al., 2011; Kamijo and Masaki, 2016), cognitive flexibility (Westfall et al., 2018), and working memory (Scudder et al., 2014) indexed by shorter response time and/or higher response accuracy. While classic theories presume that cognitive processes and development are universal, there is growing evidence that they systematically differ as a function of cultural variation and individual differences in relation to cultural diversity (Masuda and Nisbett, 2001; Nisbett et al., 2001). Cultural variations in parenting styles, educational practices, and language can lead to differences in children's cognitive processes and development (Gutchess and Rajaram, 2022). Additionally, differences in values, beliefs, and daily practices can affect how individuals process information and make decisions, which can also influence the results of research studies (Senzaki et al., 2018). Given the diverse childcare practices across Arab, European, and Asian countries, it is also challenging to generalize the physical activity and fitness of Arab children compared to their peers. Factors such as access to outdoor spaces, changing social norms, and urbanization can affect children's activity levels and thus their aerobic fitness. Climate may further play a role in children's physical activity and fitness, especially in the Middle East, where hot weather is prevalent most of the year. The huge cultural differences between the Western, Asian, and Arab world limit the generalizability of research findings regarding the association between aerobic fitness and cognition. Therefore, the current study is the first to target the Arab world and aimed to investigate the association between aerobic fitness and cognitive functions.

We used the Attentional Network Test (ANT) to assess children's cognitive functions in the present study. Attentional networks are a framework that describes three different systems of attention: alerting, orienting, and executive control (Posner and Petersen, 1990; Petersen and Posner, 2012). The ANT is a cognitive task that measures the efficiency of these three systems. The alerting system is responsible for maintaining a state of readiness for processing incoming information. The ANT assesses this by measuring the reaction time to a sudden onset cue without a cue/warning signal. The orienting system is responsible for directing attention to specific locations in space. The ANT assesses this by measuring the reaction time to a cue that indicates the location of the target. The executive system is responsible for controlling attentional resources. The ANT assesses this by measuring the interference caused by incongruent distractors, which require the participant to inhibit an automatic response and instead respond according to a specific target. Thus, ANT is considered a reliable tool to measure the efficiency of these three different attentional systems (Brassell et al., 2017) and examine whether childhood fitness is generally or specifically associated with each aspect of the attentional networks. For example, using the ANT task, Pérez et al. (2014) examined the association between self-reported physical activity and three attentional networks in Spanish young adults, finding that higher-active individuals exhibited superior task performance on the executive but not altering and orienting networks. Given that this study is the first study to examine the association between fitness and cognition in Arab children, the ANT that includes three distinct aspects of cognition is considered to be suitable to investigate whether previous findings from different cultures can be generalized to Egyptian children.

The present study was designed to examine the association of aerobic fitness with the efficiency of alerting, orienting, and executive networks in Egyptian preadolescent children. Based on the abovementioned Western and Asian literature, we predicted that higher aerobic fitness would be associated with better ANT task performance indexed by shorter response time and/or greater accuracy. It was also predicted that if the positive association between aerobic fitness and cognitive function could be generalized regardless of cultural context, the association would be stronger for the executive network.

## 2. Methods

### 2.1. Participants

*A priori* power analysis was conducted using G^*^power 3.1.9.6 (Faul et al., 2007), with an α level of 0.05 and a power of 0.90, to estimate the appropriate sample size. The analysis was based on data from the previous meta-analytic review by Smith et al. (2010), which indicated a small to moderate positive association between aerobic fitness and cognitive performance (*r* = 0.26). The results of the analysis indicated that a sample of 123 participants would be required. A convenience sample of children enrolled in a primary private school in Assiut City, Egypt was recruited through advertisements on the school's bulletin boards. The following eligibility criteria were applied: children between the ages of 9 and 11 who agreed and had signed assent forms from their parents. Exclusion criteria included physical or intellectual disability and clinical, neuromotor, psychological, or cognitive contraindications (as confirmed by the school's pedagogical staff). Participants who did not complete the fitness or cognitive test were also excluded. Although 125 children were eligible for our study, data from 22 children were missing from the results because they did not complete the task (*n* = 4) due to computer failure (*n* = 6), failed to attend one of the experiment days (*n* = 8), or accuracy was below 50% (*n* = 4). Therefore, 103 participants (9.76 ± 0.11, 5.8% overweight) were included in the final analysis. Table 1 presents demographic information and fitness data for our sample. Before collecting data, legal guardians reported that their children had no neurological diseases or physical disabilities and had a normal or corrected-to-normal vision. They also reported that their children received no special education services related to cognitive or physical issues. All participants and their legal guardians properly filled and signed informed assent/consent in accordance with the Institutional Review Board at the Faculty of Physical Education, Assiut University.

**Table 1:** Demographic, aerobic fitness, and cognitive task performance measures.

	**All participants (*n* = 103)**	**Girls (*n* = 47)**	**Boys (*n* = 56)**
Age (yrs)	9.76 ± 0.11	9.64 ±0.15	9.84 ± 0.14
Height (m)	1.38 ± 0.01	1.38 ± 0.01	1.39 ± 0.01
Weight (kg)	35.92 ± 0.98	34.91 ± 1.45	36.74 ± 1.33
BMI (kg/m^2^)	18.52 ± 0.36	18.15 ± 0.52	18.82 ± 0.50
Tanner	1.34 ± 0.06	1.30 ± 0.09	1.37 ± 0.09
Grade	3.92 ± 0.08	3.85 ± 0.13	3.98 ± 0.11
Maternal education	3.64 ± 0.08	3.65 ± 0.12	3.63 ± 0.11
ADHD	6.14 ± 0.13	6.02 ± 0.20	6.23 ± 0.18
6-min running test (m)	985.50 ± 16.95	945.02 ± 24.80^*^	1018.16 ± 22.44
Congruent RT (*msec*)	835.77 ± 14.81	857.67 ± 21.97	818.11 ± 19.92
Incongruent RT (*msec*)	978.93 ± 21.86	1028.92 ± 34.73^*^	938.59 ± 26.95
Congruent accuracy (%)	93.72 ± 1.12	92.93 ± 1.83	94.35 ± 1.38
Incongruent accuracy (%)	79.79 ± 2.43	73.03 ± 4.36^*^	85.25 ± 2.44
Alerting network	47.57 ± 8.36	50.64 ± 14.45	45.09 ± 9.70
Orientation network	51.57 ± 7.03	42.69 ± 11.45	58.74 ± 8.69
Executive network	143.16 ± 11.87	171.25 ± 21.27^*^	120.49 ± 12.24

Maternal education was tested using a five-point scale ranging from 1, which indicates parents did not complete high school, to 5, which indicates that they had earned a postgraduate degree; Tanner scales refer to pubertal timing; BMI, body mass index; ADHD, scores on the ADHD rating scale IV; values are means ± SEMs; ^*^Significantly different from boys, *p* < 0.05 (*t*-tests).

### 2.2. Aerobic fitness

The 6-min run test is a simple and commonly used assessment of an individual's aerobic capacity (Bös and Mechling, 1985; Bös et al., 2021). It is part of the international physical performance test profile recommended by the German Association of Sport Sciences and validated for Egyptian school children (Abdelkarim et al., 2021). Children run around the baseline of the volleyball court field (marked by cones at 9 × 18 meters) as many times as possible within 6 min. The test was performed in groups of up to 10 subjects. A pace of 24 s per lap was set to establish a feeling for the rhythm of running. Children were allowed to both run and walk during the 6 min, with encouragement to run as much as possible. The remaining time was announced every minute during the run. After 6 min, the test subjects stop and sit on the ground. The distance in meters covered by each test subject within 6 min was measured. The distance was calculated from the number of laps (1 lap = 54 m) plus the distance of the last lap started. The field fitness test used to assess aerobic capacity in this study was comparable to previous studies (Scudder et al., 2014; Westfall et al., 2018).

### 2.3. Attention network test

**Figure 1:**
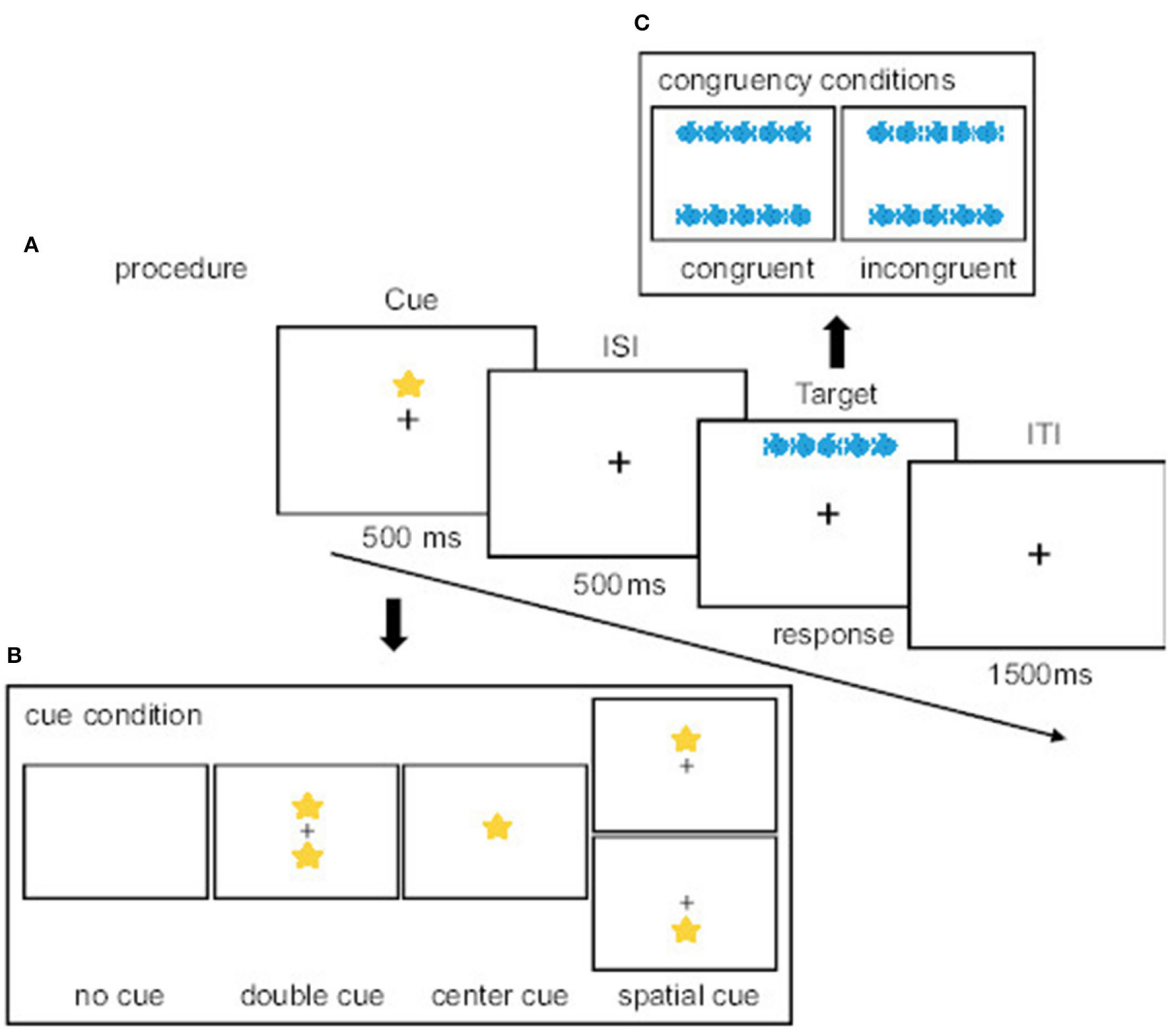
Schematic illustration of the experimental procedure including time structure of the attentional network task (ANT); **(A)** An example of the sequence of events of the experimental procedure; **(B)** Four cue conditions (no cue, central cue, double cue, and spatial cue); **(C)** Target-stimulus flanker conditions (congruent and incongruent). Icons are sourced from Microsoft's Fluent Emoji set reproduced under the terms of the MIT License.

The current study adopted a computerized version of the ANT, based on the original version (Fan et al., 2002) and modified by Chang et al. (2015) to assess the efficiency of the three dissociable attentional functions: alerting, orienting, and executive control (see Figure 1). All stimuli were presented on a computer screen at a distance of approximately 90 cm using E-prime software 3.0 (Psychology Software Tools, Pittsburgh, and PA). Target stimuli consisted of a row of five horizontal goldfish on a white background. Participants were instructed to respond with a thumb press on the keyboard to the direction of the centrally presented fish amid either congruent (e.g., 
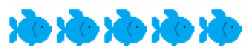
 or 
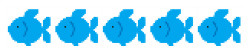
 incongruent (
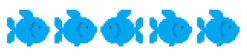
 or 
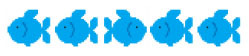
) flanking nontargets (identical fish). A cue in the form of a sea star (e.g., 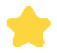) appeared at the center, both up and down locations (temporally informative), or either up or down location (spatially informative), or did not pear at all. For each trial, there were four different cue conditions: (1) no cue (no sea star before target onset); (2) double cue (both cue sea star flashes up and down locations before target onset); (3) center cue (one cue sea star flashes before target onset at the center of the screen); and (4) spatial cue (one cue sea star flashes up or down before target onset). The alerting network was assessed via the performance (e.g., response time) difference between no-cue and double-cue conditions. The orienting network was assessed via the performance difference between center cue and spatial cue conditions. The executive network was assessed via the performance difference between incongruent and congruent conditions. For more details on the three attentional networks, see Fan et al. (2002). Task instructions and encouragement to participants before and following each task block emphasized responding as quickly and as accurately as possible. The participant's response cleared the screen for the next trial; otherwise, the response stimulus remained on the screen for a maximum of 1600 ms with an inter-trial interval (ITI) of 1500 ms. The frequency of the cue and flanking conditions and their combinations were equal. All trial combinations were randomly presented in 2 blocks of 64 trials each. The entire test lasted < 10 min.

### 2.4. Procedure

After obtaining informed consent, we measured participants' standing height and weight while they were wearing lightweight clothing and no shoes. Height and weight measurements were taken using a FullMedi scale (Full Medical Co., Ltd., China). Participants' legal guardians completed health history and demographics questionnaires, including adapted Arabic versions of the ADHD Rating Scale-IV (DuPaul et al., 1998; Hassan et al., 2009), the Tanner Staging System questionnaire (Taylor et al., 2001), and Physical Activity Readiness Questionnaire (PAR-Q; Thomas et al., 1992) to check for any symptoms of ADHD, pubertal timing, and any previous health problems that might be evoked or exacerbated by the fitness test, respectively. All questionnaires were reliable and adapted for Arabic native speakers. This is because socioeconomic status has been shown to be related to both cognitive control (Mezzacappa, 2004) and fitness (Freitas et al., 2007), and maternal educational attainment was assessed as a proxy for socioeconomic status (Stevens et al., 2009). Participants were seated in the digital media lab at school and performed the ANT. They were given instructions and engaged in practice trials before the start of testing. We ensured that the participants were not involved in any physical activities before testing. The fitness battery was performed on a different day. Participants received a gift card and a small toy or a children's book.

### 2.5. Statistical analysis

We tested the efficiency of the ANT in our study by testing the basic ANT effects using a 4 (Cue condition: no cue, center cue, double cue, and spatial cue) × 2 (Flanker condition: congruent and incongruent) repeated-measures ANOVA that was separately submitted on the mean response time and response accuracy with the flanking conditions. The partial eta square was reported as the effect size for the main effect.

Pearson correlations were conducted to assess bivariate relationships between aerobic fitness and ANT performance outcomes. Next, to determine which covariates were associated with fitness and, therefore, may have influenced our outcome of interest, we performed bivariate correlations between fitness, age, sex, body mass index (BMI), and SES. Partial correlations included sex and age as control variables as these were correlated with aerobic fitness (6-min running distance). The significance level was set at *p* < 0.05. All statistical analyses were conducted using SPSS version 25.0 (IBM, Corp., Armonk, NY, USA).

## 3. Results

### 3.1. Sample characteristics

In total, 22 participants were excluded during the study, and a total of 103 participants were included in the final analysis. Demographic and aerobic fitness data are presented in Table 1.

### 3.2. Efficiency of the three attentional networks

Analysis of response time revealed a significant main effect for cue condition, *F*_(1, 102)_ = 52.14, *p* < 0.01, = 0.338, indicating that no cue exhibited the longest response time (933.46 ± 187.25 ms), followed by center and double cues (905.79 ± 169.20 and 885.88 ± 158.85 ms), and spatial cue exhibited the shortest response time (854.22 ± 184.57 ms). A main effect for the flanking condition was observed, *F*_(1, 102)_ = 161.11, *p* < 0.01, $\eta_{p}^{2}$ = 0.612, indicating that the response time was shorter in the congruent condition relative to the incongruent condition (835.77 ± 150.34 vs. 978.93 ± 221.90 ms). No interaction was observed between cue and flanker conditions (*p* = 0.27). Analysis of response accuracy revealed a main effect for the flanking condition, *F*_(1, 102)_ = 52.19, *p* < 0.01, $\eta_{p}^{2}$ = 0.338, indicating that the response accuracy was lower in the incongruent condition relative to the congruent condition (79.80 ± 24.68 vs. 93.72 ± 11.34 %). No main effect of cue condition or interaction was observed between cue and flanker conditions (*p* ≥ 0.09). The presentation of the ANT task characteristic indicates that our modified ANT task was appropriately manipulated and examined the three attentional networks.

### 3.3. Aerobic fitness and ANT task performance

Descriptive statistics regarding aerobic fitness and ANT task performance are reported in Table 1. Unadjusted bivariate correlations between aerobic fitness and behavioral accuracy yielded a significant negative correlation in the response time of the incongruent condition (*r* = −0.29, *p* < 0.01), a positive correlation in the response accuracy of the incongruent condition (*r* = −0.25, *p* < 0.05), and a significant negative correlation in the executive network (*r* = −0.32, *p* < 0.01). No significant correlation was found in response time and accuracy of the congruent condition, and attentional and orienting networks (*r*s > 0.16, *p*s > 0.09).

**Figure 2:**
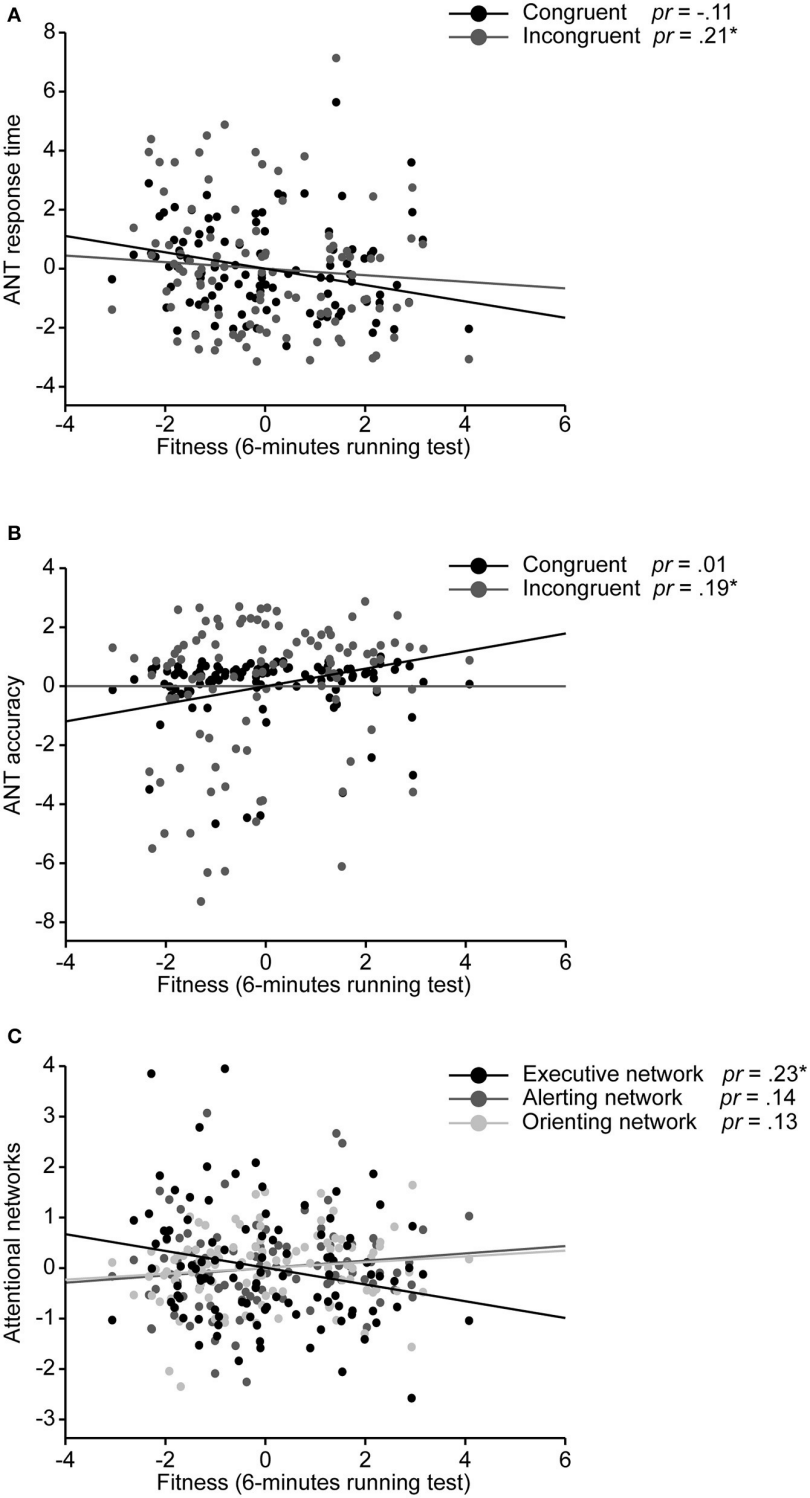
Partial regression plots depicting the relationship between aerobic fitness and ANT task performance for **(A)** congruent and incongruent response time, **(B)** congruent and incongruent response accuracy, and **(C)** three attentional networks, after controlling for age and sex. Partial correlations (*pr*) are provided. *p < 0.05.

Aerobic fitness was found to be significantly related to the control variables age (*r* = −0.36, *p* < 0.01) and sex (*r* = −0.21, *p* < 0.05), but not with BMI, maternal education, and pubertal timing (*p* ≥ 0.17). A partial correlation including age and sex as control variables continued to show a significant negative relationship between aerobic fitness and response time in the incongruent condition (*r* = 0.21, *p* < 0.05), a significant positive correlation in the response accuracy of the incongruent condition (*r* = −0.20, *p* < 0.05), and a significant negative correlation in the executive network (*r* = −0.22, *p* < 0.05; see Figure 2). The partial correlation between aerobic fitness and response time and accuracy in the congruent condition, as well as in the attentional and orienting networks, remained non-significant (*r* = 0.12, *p* ≥ 0.23).

## 4. Discussion

The current study aimed to extend the literature on aerobic fitness and cognition to the Arab world by assessing the attentional networks in Egyptian preadolescent children. As we could not achieve the required sample size, we performed a sensitivity analysis based on the current sample size of 103 participants with an α level of 0.05 and a power of 0.85 to show the robustness and reliability of the study's findings. The analysis indicated that the current study design theoretically had sufficient sensitivity to detect the independent contribution of aerobic fitness if it exceeded an effect size of *r* = 0.16. The main findings revealed that higher aerobic fitness was related to shorter response time and superior accuracy in the incongruent condition. Furthermore, greater aerobic fitness was positively associated with the executive network, while no association was observed in the attentional and orienting networks. These findings extend the extant literature in Western and Asian populations and suggest that the association between aerobic fitness and cognitive control could be generalized to Arab children.

Our data indicate that higher aerobic capacity is associated with superior performance in the ANT task, as indexed by shorter response times and higher response accuracy in the most cognitively demanding task condition (i.e., incongruent condition) and is selective for the executive network. The present study replicates previous findings in Western and Asian populations, which showed that higher-fit children exhibit disproportionately shorter response times (Chaddock et al., 2010) and greater response accuracy (Pontifex et al., 2011; Voss et al., 2011; Kamijo and Masaki, 2016) during task conditions that require the upregulation of inhibitory control (e.g., incongruent flanker condition). Furthermore, the selective nature of the relationship between fitness and the executive network is consistent with Pérez et al. (2014) who used the ANT for Spanish young adults. It is worth noting, however, that several studies showed a general positive association between aerobic fitness and cognitive performance irrespective of cognitive demands (Buck et al., 2008; Hillman et al., 2009; Aly and Kojima, 2020). Given that the same American group showed both general and selective associations (Buck et al., 2008; Hillman et al., 2009; Chaddock et al., 2010; Pontifex et al., 2011), the general vs. selective nature of the relationship between fitness and cognition during development is unlikely to be due to cultural differences. The observed selective relationship is probably due to the ceiling effect or high levels of baseline performance (Ishihara et al., 2021) and/or the selective nature of the association between aerobic fitness and cognition in some circumstances (Colcombe and Kramer, 2003). Interestingly, our findings were inconsistent with those of a recent study conducted among Brazilian children, which indicated that aerobic fitness was not related to the three attentional networks of the ANT (Cabral et al., 2021). Although the study recruited a sufficient sample size and considered several potential moderators (e.g., age, gender, and BMI), other factors such as culture, lifestyle habits, regional nutrition, or even genetic variation among different geographic regions may moderate this association. Therefore, cross-cultural studies are needed to understand how these factors may relate to changes in cognition associated with physical fitness, especially since there is a lack of evidence on this topic.

Although the aim of this study was not to elucidate the underlying mechanisms of the relationship between aerobic fitness and executive networks, one possible explanation for this association is related to the effects of aerobic exercise on brain function. Research suggests that aerobic exercise increases certain chemicals in the brain, such as brain-derived neurotrophic factor and insulin-like growth factor 1, which promote neurogenesis and improve cognitive performance (Vaynman et al., 2004; Alkadhi, 2018). Additionally, studies have shown that aerobic exercise elevates the release of neurotransmitters such as dopamine, which is essential for attention and memory (Heijnen et al., 2016). Furthermore, aerobic exercise can increase blood flow to the brain and enhance the development of the prefrontal cortex, a key area of the brain involved in cognitive control (Pereira et al., 2007; Querido and Sheel, 2007). Research in children and young adults has also indicated that aerobic fitness and a physically active lifestyle are associated with increased efficiency of brain activity (Kamijo and Masaki, 2016; Aly and Kojima, 2020). Therefore, it is believed that the combination of these changes in the brain's chemistry, structure, and function is behind the association between aerobic fitness and cognitive function.

While this study is the first to investigate the association between aerobic fitness and cognitive functions in preadolescent children in the Arab region, there are some limitations to this study that should be considered. First, as this study is observational and a cross-sectional study, it cannot determine causality. Further research should be conducted to investigate the effect of fitness on cognitive performance using long-term exercise programs in Arab societies similar to that conducted in Western (Davis et al., 2011; Castelli et al., 2020) and Asian countries (Ishihara and Mizuno, 2018; Watanabe et al., 2019) to generalize the influence of exercise on cognition in the Arab world. Second, we only focused on aerobic capacity which limits our results for other fitness components. In a recent study by Lin et al. (2022), the association of motor ability, muscular fitness, and aerobic fitness to working memory was investigated in Taiwanese school-aged children. The results showed that greater aerobic fitness was associated with smaller variations in reaction time, while both greater muscular fitness and motor ability were associated with higher response accuracy. Importantly, the positive association between motor ability and response accuracy remained significant even after controlling for muscular fitness, indicating the independent contributions of motor ability to cognitive function. These findings suggest that health-related fitness domains may have differential associations with cognitive function, and thus further research is needed to better understand the interplay between different fitness components and their relationship with cognitive outcomes in children. Third, the convenience sampling method and the relatively small sample size were other limitations of this study. A priori power analysis indicated that a sample of 123 participants was required. Unfortunately, we could not reach that sample size target due to missing data from 22 participants. To overcome this limitation, we performed a sensitivity analysis, which indicated that our study was sensitive enough to detect the association between aerobic fitness and cognitive performance. Since our study yielded positive findings for this association, it is more likely that the difference between the initially computed sample size and our final sample size did not impact our results. Future studies should utilize a more robust sampling method and use a sufficient sample size to ensure a more representative sample. Another limitation was the potential impact of confounding variables, such as nutritional habits and physical activity levels, on the association between aerobic fitness and cognitive performance (Masoomi et al., 2020). Future studies could control these variables by administering questionnaires or conducting objective measurements to assess participants' nutritional habits and physical activity levels. Lastly, examining a more varied group of participants using several cognitive tasks tapping on other aspects of cognition (e.g., memory and language-related cognition) is highly needed in the Arab region to gain a deeper understanding of the association between aerobic fitness and cognitive health. Cross-culture studies are also needed to delineate whether or not this association can be impacted by cultural differences.

## 5. Conclusion

The present study provides evidence regarding the association between aerobic fitness and cognitive control in Egyptian preadolescent children. Specifically, our findings indicate that aerobic fitness is positively associated with the executive network, while no associations were observed in the alerting and orienting networks. Moreover, the association was selective for a more cognitively demanding task condition, suggesting the selective nature of the fitness–cognition relationship in Egyptian children. To the best of our knowledge, no previous studies have examined this relationship in the Arab world. Thus, our findings among Egyptian children are comparable to previous research in Western and Asian countries, indicating that cultural variations may not alter the relationship significantly between aerobic fitness and cognition, but they may still have an impact. The present study supports the notion that aerobic fitness may play a significant role in enhancing cognitive functioning in preadolescent children, particularly in the executive network. These findings have important implications for parents, educators, and policymakers concerned with promoting the health and cognitive development of children in Egypt and beyond.

## Author's note

The icons reproduced in this study are sourced from Microsoft's Fluent Emoji set reproduced under the terms of the MIT License.

## Data availability statement

The raw data supporting the conclusions of this article will be made available by the authors, without undue reservation.

## Ethics statement

The studies involving human participants were reviewed and approved by the Research Ethics Committee, Physical Education, Assiut University. Written informed consent to participate in this study was provided by the participants' legal guardian/next of kin.

## Author contributions

MA and KK: conceptualization. OA, MA, NE, and AS: data curation and investigation. OA and MA: formal analysis and writing—original draft. OA, MA, NE, AS, AW, and KB: methodology. AW and KB: project administration. KK, AW, and KB: resources and supervision. MA, KK, AW, and KB: writing—reviewing and editing. All authors contributed to the article and approved the submitted version.
